# Lower-Neck Sparing Using Proton Therapy in Patients with Uninvolved Neck Nasopharyngeal Carcinoma: Is It Safe?

**DOI:** 10.3390/jcm11123297

**Published:** 2022-06-09

**Authors:** Francesca De Felice, Alessandro Vai, Anna Maria Camarda, Nicola Alessandro Iacovelli, Ester Orlandi

**Affiliations:** 1 Radiation Oncology, Policlinico Umberto I, Department of Radiological, Oncological and Pathological Sciences, “Sapienza” University of Rome, Viale Regina Elena 326, 00161 Rome, Italy; francesca.defelice@uniroma1.it; 2 Radiation Oncology, Clinical Department, National Center for Oncological Hadrontherapy (CNAO), Strada Campeggi 53, 27100 Pavia, Italy; alessandro.vai@cnao.it (A.V.); annamaria.camarda@cnao.it (A.M.C.); 3 Radiation Oncology Department, Fondazione IRCCS Istituto Nazionale dei Tumori di Milano, Via G. Venezian 1, 20133 Milan, Italy; nicolaalessandro.iacovelli@istitutotumori.mi.it

**Keywords:** proton, photon, IMRT, head neck cancer, nasopharyngeal carcinoma, prophylactic neck irradiation, unilateral neck irradiation

## Abstract

Undifferentiated carcinoma of the nasopharynx (NPC) is a rare disease, which usually occurs in the Asian population. Due to its anatomic location, it is characterised by rich lymph node drainage and has a high incidence of cervical node metastasis. However, cervical nodal metastasis commonly involves retropharyngeal nodes and level II nodes, followed by level III nodes. In recent years, innovations in terms of systemic treatments and radiotherapy techniques have improved oncological outcome and treatment-related toxicities. Therefore, there is a growing interest in de-intensification strategies of reducing volumes and treatment-related side effects, especially in patients with NPC with N0–N1-stage disease. Proton therapy could represent a valid alternative to Intensity Modulated Radiotherapy (IMRT) in the management of NPC in this setting. With this Commentary, we aim to explore the feasibility of Intensity Modulated Proton Therapy (IMPT) in upper-neck irradiation of NPC N1-stage disease. We selected an NPC patient with N1 disease and compared the original IMRT plan with the IMPT plan in terms of dosimetric parameters. IMPT offers a minimal dosimetric advantage over IMRT in the bilateral lower-neck sparing. Clinical trials are needed to evaluate the significance of these proposed suggestions and their applicability in non-endemic areas.

Undifferentiated carcinoma of the nasopharynx (NPC) is a unique disease, commonly affecting Asian countries and very rarely Caucasian ones [1,2]. Among others risk factors, Epstein–Barr Virus (EBV) is one of the most important causative factors and its DNA plasma load represents a prognostic factor, before and after treatment [3]. The nasopharynx is characterized by a high lymphatic network and NPC has, therefore, a high incidence of cervical node metastasis compared to other head and neck cancers [4]. Cervical nodal involvement follows an ordered pattern: retropharyngeal nodes (RLNs), level II nodes (LNs) being the most commonly involved, followed by level III LNs, VA, and IV, with probabilities of 44.9%, 26.7%, and 11.2%, respectively [5]. Skip metastases are considered rare in this setting [4]. It should be noted that LN metastases rarely occur out of the elective prophylactic irradiation of levels II, III, and VA in N0 patients and in patients with pathological RLNs only. Therefore, this might support the volume de-intensification strategy [6]. 

In recent years, the intensification of systemic treatment, the advent of Intensity Modulated Radiotherapy (IMRT), and diagnostic advances have improved the outcome for this disease and reduced the overall toxicity burden [3]. However, despite modern RT techniques, late radiation-related toxicities (such as hypothyroidism, dysphonia, dysphagia, and skin and soft tissue fibrosis) are still frequent and affect patients’ quality of life (QoL) [7,8]. In the view of de-escalating volumes in order to reduce toxicities, many studies, all conducted in endemic areas, have tried to answer the question whether it is necessary for patients with N0-N1 disease only to irradiate the whole-neck drainage areas (II-III-IV-V levels) with prophylactic intent [4,9]. All of them demonstrated there was no significant difference in disease-free survival and lower-neck control when reducing or omitting lower-neck irradiation field [6,10]. Recently, Tang et al. designed a non-inferiority, large-scale, multicentre, randomized phase 3 trial to assess whether elective upper-neck irradiation (UNI) of the uninvolved neck (including patients with both N0 and N1 disease) was non-inferior to standard whole-neck irradiation (WNI) in patients with NPC [11]. They showed that elective ipsilateral upper-neck irradiation (UNI) of the uninvolved neck provides similar regional relapse-free survival, with less late radiation-related toxicity compared with standard WNI in patients with N0–N1 nasopharyngeal carcinoma. The upper-neck lymphatic drainage areas included levels II, III, and VA; the lower-neck lymphatic drainage areas included levels IV and VB. Based on the absence or the presence of unilateral cervical lymph node involvement, patients in the UNI group received bilateral lower-neck sparing or contralateral lower-neck sparing, respectively. UNI treatment represents a simple and effective de-escalation strategy [11]. However, one remark could be if the dose received in UNI cases in ipsilateral, the lower-neck is likely to have a significant impact on contralateral regional disease control. 

From the view of reducing volumes and treatment-related side effects, the development of techniques capable of sparing organs at risk with the same target coverage and local control should be pursued. Nowadays, proton therapy represents a promising alternative to IMRT in the management of NPC [6]. In the wake of fewer side effects and radiation-treatment-related toxicities, intensity modulated proton therapy (IMPT) stands out in reducing the dose to the organs at risk, especially neurological structures (such as temporal lobes, optic pathways) and the oral cavity, while keeping a similar target coverage compared to IMRT [12,13]. IMPT could reduce the incidence of gastrostomy tube dependence, brain necrosis, and hypothyroidism [13]. 

In this context, perhaps we need to reframe the question. Is this high-level evidence, supporting UNI as a valid option to be considered in future treatment guidelines for NPC patients with N0–N1 stage disease, feasible in the case of IMPT delivery? 

To try to answer the question, we selected an NPC patient with N1 disease, managed at our centre, and then we generated and optimized three different plans (see Figure 1). We compared the original IMRT plan with the IMPT plan in terms of dosimetric parameters. As per Tang et al.’s trial scenarios, we compared mean radiation doses to (i) lower-neck in bilateral lower-neck sparing, (ii) contralateral lower-neck sparing, and (iii) standard WNI. In the case of contralateral lower-neck sparing, mean doses to contralateral lymph nodes neck levels, as well as to medial structures, including thyroid gland, glottic larynx, cervical oesophagus, and spinal cord, were lower for IMPT. IMPT offers a minimal dosimetric advantage over IMRT in bilateral lower-neck sparing. Based on these empiric data and on the assumption that photon dose to contralateral neck when treating unilateral target remains in a range of 30–45 Gy (a dose sufficiently high to ensure favourable clinical outcomes), our concern is about the eventuality that a lower radiation dose with proton therapy could be associated with worse local control, especially in contralateral lower-neck sparing cases. Although these dosimetric comparisons are theoretical and non-validated, they represent an attempt to better understand lower-neck sparing while maintaining excellent target coverage. Surely, clinical trials are needed to evaluate the significance of these proposed suggestions and their applicability in non-endemic areas, where EBV-related NPC is less common [14]. Pre-treatment plasma EBV-DNA values, post-induction chemotherapy plasma EBV-DNA values and circulating tumour cells values could help in stratifying patients that would benefit the most from de-escalating strategies [15,16].

## Figures and Tables

**Figure 1:**
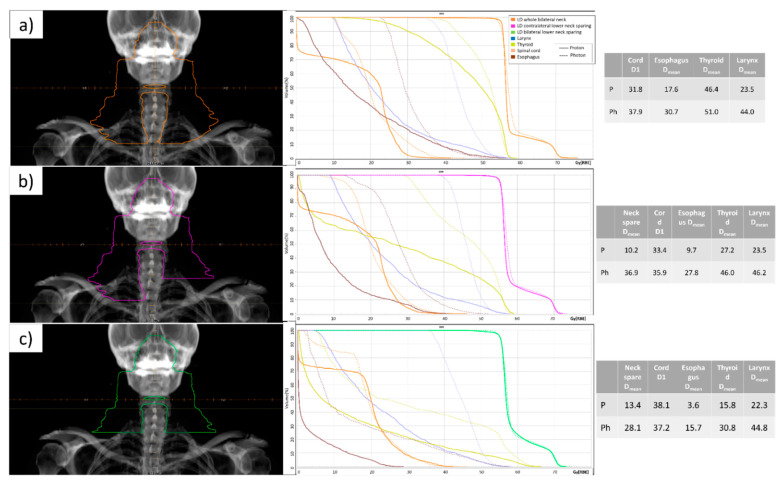
**Dose map comparison.** Low risk CTV with (**a**) whole bilateral neck; (**b**) contralateral lower-neck sparing; (**c**) bilateral lower-neck sparing according to [11]. In all cases target volumes were planned to receive 56 Gy [RBE] in 33 fractions, according to study protocol. In the three columns for each case, the following were reported, respectively: DDRs with the relative target; IMPT (continuous) versus IMRT (scattered) plans DVH for the low-dose target and selected OARs; dose values (Gy[RBE]) for the selected OARs.
